# Conflicting Actions of Inhalational Anesthetics, Neurotoxicity and Neuroprotection, Mediated by the Unfolded Protein Response

**DOI:** 10.3390/ijms21020450

**Published:** 2020-01-10

**Authors:** Hiroshi Kokubun, Hisayo Jin, Mari Komita, Tomohiko Aoe

**Affiliations:** 1 Department of Anesthesiology, Chiba University Graduate School of Medicine, Chiba 260-8670, Japan; hiro_kokubun127@yahoo.co.jp (H.K.); jinhy@chiba-u.jp (H.J.); 2 Department of Anesthesiology, Chiba Rosai Hospital, Ichihara 299-0003, Japan; mari29@kj9.so-net.ne.jp; 3 Department of Medicine, Pain Center, Chiba Medical Center, Teikyo University, Ichihara 299-0111, Japan

**Keywords:** anesthetics, chaperone, endoplasmic reticulum, ER stress, KDEL receptor, unfolded protein response, neuroprotection, neurotoxicity

## Abstract

Preclinical studies have shown that exposure of the developing brain to inhalational anesthetics can cause neurotoxicity. However, other studies have claimed that anesthetics can exert neuroprotective effects. We investigated the mechanisms associated with the neurotoxic and neuroprotective effects exerted by inhalational anesthetics. Neuroblastoma cells were exposed to sevoflurane and then cultured in 1% oxygen. We evaluated the expression of proteins related to the unfolded protein response (UPR). Next, we exposed adult mice in which binding immunoglobulin protein (BiP) had been mutated, and wild-type mice, to sevoflurane, and evaluated their cognitive function. We compared our results to those from our previous study in which mice were exposed to sevoflurane at the fetal stage. Pre-exposure to sevoflurane reduced the expression of CHOP in neuroblastoma cells exposed to hypoxia. Anesthetic pre-exposure also significantly improved the cognitive function of adult wild-type mice, but not the mutant mice. In contrast, mice exposed to anesthetics during the fetal stage showed cognitive impairment. Our data indicate that exposure to inhalational anesthetics causes endoplasmic reticulum (ER) stress, and subsequently leads to an adaptive response, the UPR. This response may enhance the capacity of cells to adapt to injuries and improve neuronal function in adult mice, but not in developing mice.

## 1. Introduction

General anesthesia is an essential medical treatment for surgery. Inhalational anesthetics provide adequate loss of consciousness and a certain degree of analgesia, and are widely used in current medial practice. The premise of general anesthesia is that it is reversible. It is widely accepted that an inhalational anesthetic loses its effect if it is excreted from the site of effect. The whole body then returns to its original state, including the nervous system. However, over recent years, it has become clear that the action of inhalational anesthetics is not necessarily reversible. For example, Jevtovic-Todorovic showed that pediatric anesthesia in 7-day-old infant rats, using midazolam, nitrous oxide, and isoflurane, caused widespread apoptotic neurodegeneration in the developing brain, and persistent cognitive impairments [1]. Subsequent studies confirmed that common inhalational anesthetics, such as isoflurane and sevoflurane, cause neuronal degeneration in the developing brain [2]. These anesthetics resulted in impaired neurogenesis, and induced delayed-onset neurocognitive dysfunction in infant rats [3]. Early exposure of infant rhesus monkeys to sevoflurane, within one month of birth, caused increased anxiety-related emotional behaviors by the time monkeys reached 6 months of age [4], and late-onset impairments in visual recognition memory by 12 months of age [5].

Retrospective human clinical cohort studies further revealed that multiple exposures to anesthetics prior to the age of 2 years [6], or 4 years [7], was a significant risk factor for subsequent learning disabilities. On the other hand, a sibling-matched cohort study (the Pediatric Anesthesia Neurodevelopment Assessment, PANDA study), exposed otherwise healthy children undergoing inguinal hernia surgery to a single dose of anesthetic prior to 36 months of age; interestingly, these children did not develop cognitive dysfunction in their subsequent childhood [8]. The General Anesthesia compared to Spinal anesthesia (GAS) trial also revealed that exposure to sevoflurane less than 1 h into infancy did not increase the risk of an adverse neurodevelopmental outcome at 2 years of age compared with awake-regional anesthesia [9]. Healthy infants therefore appear to withstand single exposure to anesthetics for a short duration.

Several laboratory studies have demonstrated the neuroprotective ability of inhalational anesthetics. For example, isoflurane was shown to reduce the proliferation of neuronal progenitors in rats on postnatal day 7, but increased such proliferation by postnatal day 60 [3]. In another study, sevoflurane was shown to reduce focal ischemic brain damage in an experimental rat model [10]. Evidence suggests that the neuroprotective effects of inhalational anesthetics, such as sevoflurane and isoflurane, are due to a reduction in the excitotoxicity of *N*-methyl-d-aspartate (NMDA) [11,12]. Exposure to sevoflurane has also been shown to improve cognitive function in both young (8–10 weeks) and aged rats (19 months) [13]. The general mechanism responsible for these conflicting effects, neurotoxicity and neuroprotection, remains unelucidated [14,15,16].

One possible factor underlying the neurotoxicity of inhalational anesthetics is a change in intracellular Ca^2+^ kinetics [17]. The endoplasmic reticulum (ER) is the main Ca^2+^ pool in cells; Ca^2+^ is released from the ER into the cytosol via the inositol 1,4,5-triphosphate receptor (ITPR), and the ryanodine receptor, on the ER membrane [18,19,20]. Inhalational anesthetics can exert influence over these Ca^2+^ channel receptors, and thus induce an aberrant rise in cytoplasmic Ca^2+^ [21]. An excess of cytoplasmic Ca^2+^ would then enter the mitochondria, thus resulting in the activation of caspases and ultimately, cell death [22,23]. Since ER molecular chaperones are Ca^2+^ binding proteins, it follows that aberrant Ca^2+^ mobilization will lead to the disruption of protein folding in the ER, thereby causing ER stress [24].

Secreted proteins, and membrane proteins, are synthesized by ribosomes on the ER membrane. These proteins are subsequently translocated to the ER membrane and undergo folding to become functional proteins with mature structures; this process involves significant interaction with molecular chaperones in the ER, including binding immunoglobulin protein (BiP). Subsequently, these proteins are transported to the secretory pathway to carry out their functional role; for example, as such as cell surface receptors or secretory proteins [24,25]. ER stresses, such as hypoxia, ischemia, malnutrition, or mutation, initiates an adaptive response referred to as the unfolded protein response (UPR) [26]. The UPR improves the capacity of cells to cope with ER stress, and do so by increasing the production of ER molecular chaperones, degrading unfolded proteins, and by repressing the synthesis of new proteins [27]. However, the capacity for the UPR to yield protective effects is limited. Extensive ER stress, that is beyond the capacity to adapt, has been shown to cause cellular dysfunction and cell death, thus leading to a range of human disorders, including cardiovascular disease [28], tumorigenesis [29], and neurodegenerative diseases [30,31,32,33].

We previously showed that exposure to inhalational anesthetics induced ER stress, leading to neuronal cell death and cognitive dysfunction in the developing brain of a knock-in mouse model expressing a mutant form of the *Bip* gene [34,35]. The BiP protein is a major ER-resident molecular chaperone that helps to fold proteins and regulate the UPR [36]. ER membrane proteins, such as protein kinase RNA (PKR)-like ER kinase (PERK, or eukaryotic translation initiation factor 2 alpha kinase 3; EIF2AK3), inositol requiring enzyme 1 (IRE1, or endoplasmic reticulum to nucleus signaling 1; ERN1), and activating transcription factor 6 (ATF6), are normally bound to BiP in the resting state [26]. Under ER stress, BiP binds to unfolded proteins and these membrane proteins become activated. IRE1 and PERK are multiplexed, and become activated by autophosphorylation [37]. IRE1 contains an endoribonuclease domain that splices X-Box Binding Protein 1 (XBP1) mRNA, and the active form of XBP1 mRNA is then translated [38]. The XBP1 protein promotes the transcription of various genes required for the UPR [39]. PERK is a serine/threonine kinase that inactivates eukaryotic translation initiation factor 2A, thereby suppressing the translation of most proteins [40]. ATF6 is transported to the Golgi complex, cleaved, and the cytosolic N-terminus acts as a transcription factor, thus facilitating the transcription of molecular chaperones, including BiP and transcriptional factors such as XBP1 and C/EBP homologous protein (CHOP) [41,42].

Wild-type BiP is secreted from the ER to the Golgi at a constant rate, while the carboxyl-terminal Lys-Asp-Glu-Leu (KDEL) sequence of BiP is recognized by the KDEL receptor in the Golgi [43]. BiP is returned back to the ER by coat protein I (COPI) vesicles [44]. Mutant BiP lacks the KDEL sequence and exhibits impairment in terms of ER function [25]. Homozygous mutant *Bip* mice die on the first day after birth because their ability to synthesize pulmonary surfactant is impaired [45]. The heterozygous *Bip* mutant mouse, however, is viable and develops late-onset neurodegenerative diseases [35,46].

In this study, we examined the relationship between the UPR and the neurotoxic and neuroprotective effects of inhalational anesthetics using *Bip* mutant mice and cultured neuronal cells.

## 2. Results

### 2.1. Exposure to Inhalational Anesthetics Induces the UPR

**Figure 1:**
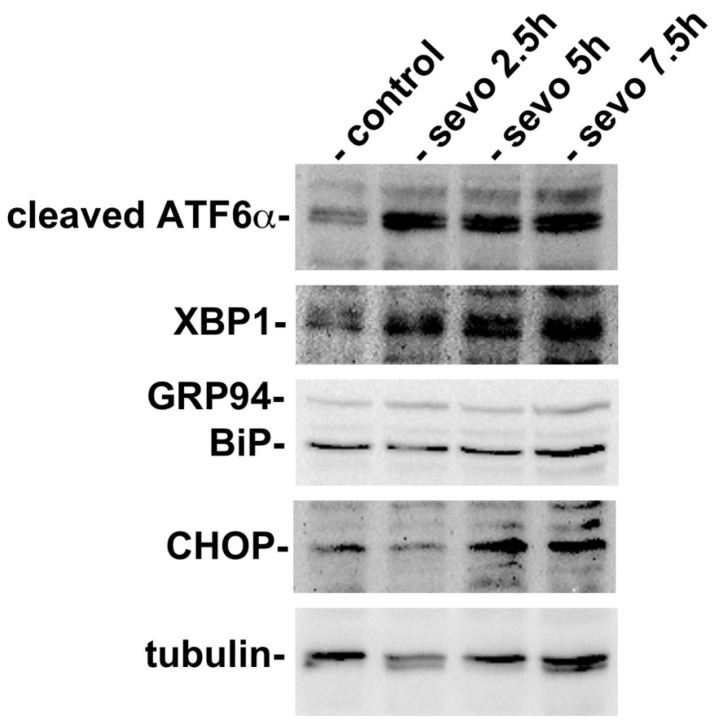
Exposure to sevoflurane resulted in the initiation of the unfolded protein response (UPR). Neuro2a cells were cultured and exposed to 3% sevoflurane for 2.5, 5, or 7.5 h. The cells were then collected and analyzed by western blotting. Sevoflurane exposure activated ATF6 and induced the expression of XBP-1, a transcription factor that enhanced the expression of BiP, a major endoplasmic reticulum (ER) chaperone. GRP94, another ER chaperone is recognized by the anti-KDEL antibody. CHOP, a transcription factor responsible for initiating apoptotic cell death during ER stress, was expressed at significantly higher levels after 7.5 h of exposure.

In order to investigate whether inhalational anesthetics influence the function of the ER at the cellular level, we exposed a mouse neuroblastoma cell line, neuro2a, to sevoflurane. Neuro2a cells were cultured at 37 °C in an atmosphere containing 21% O_2_ and 5% CO_2_. Neuro2a cells were then exposed to 3% sevoflurane for 2.5 h, 5 h, or 7 h. Subsequently, cells were collected and analyzed by western blotting for protein expression related to the UPR (Figure 1). We found that exposure to sevoflurane caused an increase in the expression of cleaved ATF6α, thus indicating that the UPR had been initiated. The expression of XBP1 and BiP was observed even under the control condition, but their expression levels increased as the length of exposure to sevoflurane increased. CHOP, a transcription factor that is expressed in response to ER stress and induces cell death [47,48], was expressed at very low levels under the control conditions, but was expressed at higher levels in cells exposed to sevoflurane. A significant increase in the expression of CHOP was observed at 7.5 h. These results suggested that sevoflurane affected cells and inhibited the protein folding of nascent proteins in the ER, thus inducing the UPR. Subsequently, the UPR enhanced the expression of cytoprotective ER chaperones, such as BiP, but also induced the expression of CHOP, thus resulting in cell death under conditions in which the ER is seriously stressed.

### 2.2. Anesthetic Exposure Caused Cognitive Impairment in the Developing Brain

**Figure 2:**
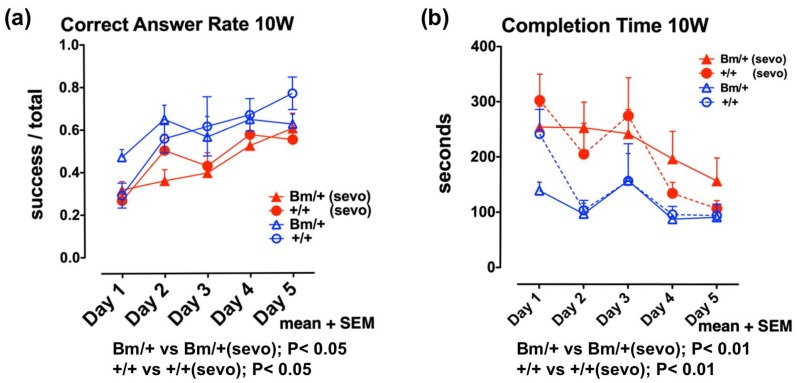
Anesthetic exposure during the perinatal period had negative effects on spatial working memory in young mice. Eight-arm radial maze testing was performed in wild-type mice (+/+; circles) and *Bip* mutant mice (Bm; triangles) at 10 weeks after sevoflurane exposure (+/+ sevo, *n* = 12, closed red circles; Bm/+ sevo, *n* = 16, closed red triangles), and without exposure (+/+, *n* = 6, open blue circles; Bm/+, *n* = 8; open blue triangles). Tests were performed for 5 days. Statistical significance was determined by one-way repeated measures analysis of variance (ANOVA) followed by Bonferroni’s multiple comparison testing. (**a**) Correct answer rates are shown as the mean value of each group + standard error of the mean (SEM). The correct answer rate was defined as the ratio of successful attempts to overall attempts (0.0–1.0). (**b**) Mean completion times for each group + SEM (data extracted from DATASET S1 and DATASET S2, *Front. Neurosci.* 12:753. doi:10.3389/fnins. 2018.00753). Fetal anesthetic exposure resulted in a lower correct answer rate (*p* < 0.05) (**a**), and a longer completion time (*p* < 0.01) (**b**), compared with unexposed mice; this was the case for both *Bip* mutant mice and wild-type mice.

Next, we investigated the effects of inhalational anesthetics on the developing mouse brain in vivo. Since the exposure of neonatal mice to inhalational anesthetics could suppress their respiration and circulation, possibly resulting in hypoxia and low blood flow, we exposed pregnant mice to sevoflurane 2 days before delivery. Female wild-type mice were mated with male heterozygous mutant *Bip* mice. On day 17.5 of gestation, pregnant mice were exposed to 3% sevoflurane for 3 h under 40% O_2_. During exposure, we measured body temperature, blood pressure in the tail, blood flow in the tail, and percutaneous oxygen saturation. Overall, we observed a reduction in blood pressure, although blood flow and SpO_2_ did not decrease. The newborns showed a ratio of 1:1 (wild-type:heterozygous mutant *Bip* mice) and were raised by the parent mice. At 10 weeks of age, we evaluated their cognitive function [35]. Radial maze testing is widely used as a spatial cognitive function test in mice [49]. Testing was performed for 5 consecutive days, during which learning effects (shorter completion time and higher correct answer rate) were observed in individual mice. Radial maze testing at 10 weeks revealed that both wild-type mice, and the heterozygous mutant *Bip* mice, exposed to anesthetic, had a significantly longer completion time (*p* < 0.01), and a lower correct answer rate (*p* < 0.05), compared with unexposed mice (Figure 2, Supplementary Materials S1).

### 2.3. Moderate Exposure to Anesthetics Causes Cytoprotective Effects through the UPR

**Figure 3:**
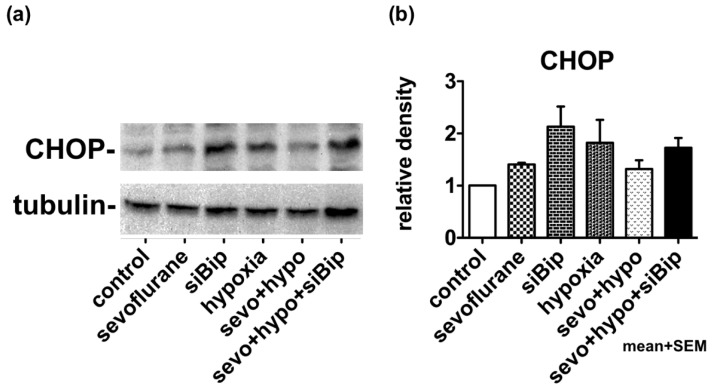
Pre-exposure to sevoflurane may induce the UPR and reduce the damage caused by hypoxia. (**a**) Cells were exposed to 3% sevoflurane for 5 h. After 12 h of recovery time, cells were exposed to hypoxic conditions (1% O_2_) for 17 h before collection. Hypoxia alone induced the expression of CHOP. Pre-exposure to sevoflurane reduced the expression of CHOP while siBiP enhanced the expression of CHOP. (**b**) Densitometry was performed for three experiments. The density of CHOP relative to that of tubulin was measured by Image J and analyzed by one-way analysis of variance (ANOVA) followed by Dunnett’s multiple comparison test. Values indicate mean + standard error of the mean (SEM). Control vs. siBip; *p* < 0.05.

We considered that exposure to inhalational anesthetic caused ER stress at the cellular level, thus inducing the expression of CHOP (Figure 1). We also considered that the UPR also induces molecular chaperones, such as BiP, that can exert cytoprotective effects. Then, we exposed neuro2a cells to 3% sevoflurane for 5 h, and returned them to normal culture conditions (37 °C, 21% O_2_, and 5% CO_2_) for 12 h, thus allowing them to compensate for the stress. Subsequently, cells were cultured for 17 h in hypoxic conditions of 1% O_2_. The expression of CHOP was then evaluated by western blotting. When cells were exposed to hypoxia alone, the expression of CHOP was induced to some extent, but in cells that had been previously exposed to sevoflurane, the expression of CHOP was clearly suppressed, thus indicating the existence of some form of neuroprotection. When the expression of BiP was suppressed by siRNA, the expression of CHOP was notably increased (*p* < 0.05.) and the neuroprotective effect of sevoflurane appeared to disappear. These results suggested that pre-exposure to sevoflurane induced the UPR, which subsequently increased the capacity of cells to tolerate hypoxic conditions via the expression of cytoprotective chaperones, such as BiP (Figure 3, Supplementary Materials S2).

### 2.4. Pre-Exposure to Sevoflurane Improves Cognitive Function in Adult Wild-Type Mice, but not in Bip Mutant Mice

**Figure 4:**
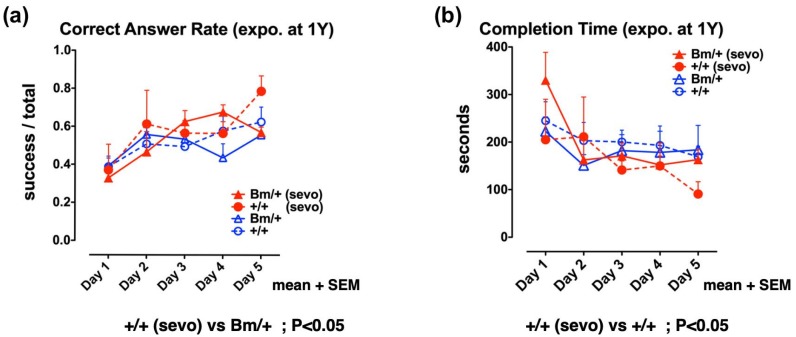
Anesthetic exposure had positive effects on spatial working memory in adult wild-type mice, but not mutant BiP mice. Eight-arm radial maze testing was performed in adult wild-type mice (+/+; circles) and mutant-*Bip* mice (Bm; triangles) 7 days after sevoflurane (sevo) exposure (+/+ sevo, *n* = 4, closed red circles; Bm/+ sevo, *n* = 5, closed red triangles) and without exposure (+/+, *n* = 7, open circles; Bm/+, *n* = 10; open triangles). Tests were performed over 5 days. Significance was determined by one-way repeated measures ANOVA followed by Bonferroni’s multiple comparison testing. (**a**) Correct answer rates are shown as the mean value for each group + standard error of the mean (SEM). The correct answer rate was defined as the ratio of successful attempts to the number of overall attempts (0.0–1.0). (**b**) Completion times represent the mean for each group + SEM.

Finally, we evaluated the effect of inhalational anesthetics on the cognitive function of adult mice. One-year old wild-type, and heterozygous *Bip* mutant mice, were exposed to 3% sevoflurane for 3 h in an atmosphere containing 40% O_2_. A radial maze test was then carried out each day from the 7th day after exposure, for a total period of 5 days. There was no significant difference in terms of cognitive function when compared between the unexposed wild-type and the mutant *Bip* mice, whereas the wild-type mice exposed to sevoflurane were associated with a significantly higher rate of correct answers when compared with unexposed *Bip* mutant mice (*p* < 0.05). In addition, the wild-type mice exposed to sevoflurane showed a significantly shorter completion time when compared with the unexposed wild-type mice (*p* < 0.05). Pre-exposure to inhalational anesthetics significantly improved cognitive function in the wild-type mice, while no such effect was evident in the *Bip* mutant mice (Figure 4, Supplementary Materials S3).

These results suggest that exposure to inhalational anesthetics may confer neurotoxic effects, thus inducing adaptive responses of UPR. The UPR enhances the capacity of cells, and individuals, to tolerate such insults. This scenario applies to healthy wild-type individuals, but not sensitive individuals, such as neonates with developing neuronal cells, and aged individuals with degenerative neuronal cells (Figure 2 and Figure 4).

## 3. Discussion

The exposure to inhalational anesthetics has neurotoxic effects, the extent of which depend on the duration and dose. However, moderate levels of exposure many have beneficial effects on appropriate subjects. The exposure to inhalational anesthetics appears to exert a pre-conditioning function. The concept of preconditioning was conceived some time ago [50]. For example, in cardiac surgery, it is now well known that a period of moderate ischemia can cause preconditioning and thereby enhancing resistance to a subsequent period of ischemia over a longer period [51]. It has also been shown that ischemic preconditioning is effective not only in the heart [52] but also in the nervous system [53], skeletal muscle [54], and liver [55]. The mechanism underlying these beneficial effects is thought to be related to changes in the expression of various genes in response to moderate preconditioning, including the production of heat-shock proteins [56], and the ER molecular chaperone, BiP [57]. Injuries that perturb protein folding in cells can induce a heat shock response in the cytoplasm, and the UPR in the ER, thus increasing the production of cytoprotective molecular chaperones, including heat shock protein 70 (HSP70) and BiP. Consequently, cells gain enhanced resistance to further cytotoxic injuries. This integrated form of stress response is involved in a variety of pathological conditions [58,59]. Other research has shown that during anesthesia for cardiac surgery, inhalational anesthetics produce similar effects as those induced by ischemia [60]. Thus, sevoflurane has been used as a clinically beneficial agent for anesthetic preconditioning, or pharmacological preconditioning [61]. Inhalational anesthetics act on cells throughout the whole body, and the phenomenon observed in the myocardium is thought to occur in all tissues, including the nervous system. In fact, several studies have reported the neuroprotective effects of anesthetic preconditioning [62,63,64,65].

In this study, we showed that exposure to sevoflurane induces the UPR; this concurred with our previous findings [34]. The UPR is initiated by the activation of ATF6, PERK, and IRE1 [26]. Distinctive roles of these three pathways are suggested by analysis of their gene knockout mice [66]. We assessed the initiation of the UPR induced by anesthetic exposure with western blot analysis of ATF6α and XBP1. Since XBP1 protein are translated after splicing of XBP1 mRNA by activated IRE1 [38], the detection of XBP1 protein represents the activation of IRE1. ATF6α and ATF6β double knockout mice are embryonic lethal [67]. XBP1 knockout mice are embryonic lethal [68]. IRE1α knockout mice are also embryonic lethal [69]. While PERK knockout mice develop diabetes mellitus, they are viable [70]. Although the PERK pathway was not evaluated in this study, the detection of cleaved ATF6α and XBP1 may have been able to assess, if not completely, the initiation of the UPR.

The UPR can enhance the resistance of cells to injuries. However, sevoflurane can also cause ER stress, and can eventually induce cell death if the exposure is excessive. The developing brain is highly sensitive to injuries. Moreover, it is thought that certain types of cells in the same individual are highly sensitive, such as actively dividing stem cells. If important neuronal cells suffer from a failure to migrate, or die, then the consequences of these effects may later manifest as cognitive dysfunction or higher brain damage, although it might not be immediately apparent. A previous study, involving the rat model, reported that inhalational anesthetics could disturb the neuronal migration in the developing brain [71]. Fetal isoflurane exposure was also shown to diminish the expression of reelin, a large glycoprotein secreted by neurosecretory Cajal-Retzius cells and responsible for mediating cortical neuronal migration in the developing brain [72]. The dysfunction of reelin has been suggested to cause higher brain disorders, such as autism [73]. In our previous paper, homozygous *Bip* mutant mice showed impaired neuronal migration [74]; the production of reelin was shown to be diminished in the Cajal-Retzius cells, while transcription of the *reelin* gene was preserved. The dysfunction of BiP in the *Bip* mutant neonates may affect the folding of large secretory proteins, such as reelin. The protein folding environment in the ER is highly sensitive to injuries, including ischemia, hypoxia, toxic substances, and malnutrition. Thus, the UPR may represent one of the most important cellular adaptive mechanisms to injury. We were the first to report that fetal exposure to inhalational anesthetics causes ER stress and neuronal cell death [34]; since then, several other papers have confirmed that general anesthesia causes ER stress [75,76,77,78,79,80,81].

In our previous study, the fetal exposure of homozygous *Bip* mutant mice to 3% sevoflurane for 3 h resulted in neuronal cell death, but not in the heterozygous mutant or the wild-type mice [34]. After delivery, both the heterozygous mutant, and wild-type mice, appeared to be normal, despite the fact that they had been exposed to sevoflurane during their time as a fetus. However, further analysis showed that these mice suffered from cognitive dysfunction when examined 10 weeks after birth. In contrast, the same level of exposure (3% sevoflurane for 3 h) resulted in a slight improvement in cognitive function when tested 7 days after exposure in adult wild-type mice, but not the heterozygous *Bip* mutant. The overall outcome of anesthetic exposure appears to depend on the balance between the extent of injury, and the sensitivity of the subjects. In the heterozygous *Bip* mutant mice, cognitive function tended to decline faster than in the wild-type mice. However, the cognitive function of wild-type mice also fades with advanced age; thus, there is no significant difference between the aged wild-type and mutants [35]. Cognitive function is superior in young mice, and aging exerts more of an influence than genotype. The exposure of fetuses to sevoflurane results in a decline of cognitive function, regardless of genotype; however, when adults were exposed, wild-type mice showed slight improvements in cognitive function. No such changes in cognitive function were observed in the *Bip* mutant mice (Figure 4).

There is no clear evidence, from either animal experiments or clinical studies, that sevoflurane anesthesia subsequently leads to poorer cognitive function. One study showed that one minimum alveolar concentration (MAC) of sevoflurane exposure led to an improvement in the cognitive function of 10 week-old rats and 20 month-old rats [13]. In another study, using APP23 Alzheimer’s disease transgenic mice expressing mutant amyloid-β precursor protein, and wild-type control mice, exposure to one MAC of isoflurane for 2 h led to an improvement in cognitive function in both types of mice. Regardless of genotype, young mice (4 months-old) were superior in terms of cognitive function when compared with aged mice (14–16 months-old) [82].

Several clinical studies have also reported the beneficial effect of inhalational anesthetics in terms of post-operative cognitive function; however, other studies have reported an increased risk for post-operative cognitive decline. In a randomized, controlled trial involving patients undergoing on-pump cardiac surgery, sevoflurane anesthesia showed better results in terms of a cognitive function test on days 2–6 post-surgery, when compared with propofol anesthesia, [83]. In another study, propofol anesthesia was reported to exert less cognitive impairment one week after surgery than sevoflurane anesthesia when patients over 65 years of age underwent cancer surgery for more than 2 h [84]. Another study described the postoperative cognitive function of twin surgical patients, but only reported a slight effect with regards to surgical anesthesia; cognitive function was reduced slightly following major surgery, and showed slight improvements following hip and knee surgery [85]. A retrospective cohort study of patients with mild cognitive impairment (MCI) and healthy controls aged 70–89 years, showed that MCI was not related to general anesthesia at ages over 40 years [86]. In a prospective cohort study of people over 65 years of age free of dementia at baseline, there was no relationship between anesthesia experience and the incidence of dementia or Alzheimer’s disease; people who underwent less invasive forms of surgery had a lower incidence of dementia than people with no history of anesthesia [87]. Another study compared patients over 50 years-of-age who had been newly diagnosed with dementia with a control group; those who had experienced general anesthesia had a higher incidence of dementia. In addition, people with a history of diabetes, hypertension, or cerebral infarction, were also shown to have a higher incidence of dementia [88].

Inhalational anesthetics are associated with longer exposure times; furthermore, the higher the exposure concentration, the greater the effect. It is difficult to determine the specific concentration and duration to use for preconditioning. Sensitivity is known to vary from individual to individual; it also appears that sensitivity varies according to cell type, and even within the same individual. The degree of invasion that is appropriate for preconditioning therefore shows individual variation and may also depend on the patient’s condition at the time of exposure.

## 4. Materials and Methods

### 4.1. Cells and Reagents

The murine neuroblastoma cell line, neuro2a, were cultured with a complete medium that consisted of Dulbecco’s modified Eagle’s medium (DMEM; Sigma Chemical, Saint Louis, MO, USA) with 10% fetal bovine serum, 2 mM glutamine, 50 µg/mL streptomycin, and 50 U/mL penicillin G at 37 °C in a 5% CO_2_ incubator. For hypoxic experiments, N_2_ was injected into the incubator to create a 1% oxygen concentration. The following antibodies were used during our experiments: Mouse mAb against γ-tubulin (Sigma Chemical, Saint Louis, MO, USA), mouse mAb SPA-827 against BiP (KDEL sequence; Stressgen, Victoria, BC, Canada), rabbit antiserum against CHOP/GADD153 (Santa Cruz Biotechnology, Dallas, TX, USA), mouse mAb 37-1, 73–505 against ATF6α (Bio Academia, Suita, Japan), and mouse mAbs M-186 and sc-7160 against XBP-1 (Santa Cruz Biotechnology, Dallas, TX, USA). siRNA oligonucleotides for *Bip* (sc-35522), and control siRNA (sc-37007) were purchased from Santa Cruz Biotechnology. For siRNA uptake, neuro2a cells were transfected with siRNA oligonucleotides in accordance with the manufacturer’s instructions. At 24 h post-transfection, the medium was changed to a complete medium and cells were exposed to sevoflurane.

### 4.2. Animals

This study was carried out in accordance with the recommendations of the guidelines for animal experiments of Chiba University. The protocol (30–132) was approved by the Institutional Animal Care Committee of Chiba University, Chiba, Japan. The generation of the knock-in mouse, expressing a mutant form of BiP that lacked the carboxyl-terminal KDEL sequence, was described previously [45]. The missing KDEL sequence was replaced with a hemagglutinin (HA) tag. The heterozygous *Bip* mutant male mice (Bm/+) were mated with wild-type (C57BL/6, +/+) female mice, and offspring (wild-type and heterozygous mutant) were bred for use in experiments.

### 4.3. Inhalational Anesthetic Exposure

Adult heterozygous *Bip* mutant mice, and wild-type mice (approximately one year-of-age), were exposed to 3% sevoflurane for 3 h, as previously described [34]. Neuro2a cells were exposed to 3% sevoflurane for 2.5–7.5 h, as previously described [34]. Previous studies suggested that 3.0% sevoflurane was almost equivalent to 0.6 MAC for mice [89].

### 4.4. Eight-Arm Radial Maze Test

Mice were subjected to the eight-arm radial maze test 7 days after being exposed to sevoflurane, as previously described [35]. The completion time (in seconds) was defined as the total time taken by each mouse to eat all of the food in the eight arms of the maze. Attempts to enter arms without bait were counted as errors, while attempts to enter arms with food were counted as successes. The correct answer rate was defined as the ratio of successful attempts to the overall number of attempts (0.0–1.0). Testing was carried out daily for 5 days (Supplementary Materials S3, Figure 4). Data related to fetal sevoflurane exposure were extracted from DATASET S1 and DATASET S2, as described in our previous study (Supplementary Materials S1, Figure 2) [35].

### 4.5. Western Blotting

Cultured cells were homogenized in a buffer containing 0.4% (*w*/*v*) Nonidet P-40, 0.2% *N*-lauroylsarcosine, 10 mM Tris/HCl pH 8.0, 30 mM EDTA, 10 µg/mL aprotinin, 10 µg/mL leupeptin, and 30 µg/mL *N*-acetyl-l-leucinal-l-lecinal-l-norleucinal (ALLN, Sigma Chemical). Western blotting was then performed and analyzed with LAS-1000 and Image Gauge Software (Fuji Photo Film Co. Ltd., Tokyo, Japan), as described previously [34]. Densitometry was performed using ImageJ software (NIH, Bethesda, MD, USA).

### 4.6. Statistical Analysis

The results of the radial maze test are shown as means + standard error of the mean (SEM). Repeated measures analyses of variance (ANOVA), followed by Bonferroni multiple comparison tests, were then performed to compare values between groups (GraphPad Prism 4.0, GraphPad Software, San Diego, CA, USA). Statistical significance was accepted at *p* < 0.05. Densitometry was carried out for the CHOP images and resultant data analyzed by one-way ANOVA, followed by Dunnett’s multiple comparison test.

## 5. Conclusions

Inhalational anesthetics cause neurotoxicity, but can also induce adaptive responses, such as the UPR in neuronal cells. Therefore, inhaled anesthetics may exert neuroprotective effects. It is therefore necessary to use inhalational anesthetics in an appropriate manner, depending on the patient’s individual condition.

## Supplementary Materials

Supplementary Materials can be found at https://www.mdpi.com/1422-0067/21/2/450/s1.

## Abbreviations

ER	Endoplasmic reticulum
UPR	Unfolded protein response
BiP	Binding immunoglobulin protein
KDEL	Lys-Asp-Glu-Leu
COPI	Coat protein I
CHOP	C/EBP homologous protein
PERK	Protein kinase RNA (PKR)-like ER kinase
IRE1	Inositol requiring enzyme 1
ATF6	Activating transcription factor 6
XBP1	X-box-binding protein 1
NMDA	N-methyl-D-aspartate
ITPR	Inositol 1,4,5-triphosphate receptor
MAC	Minimum alveolar concentration
MCI	Mild cognitive impairment
